# Human vascular cell responses to the circulating bone hormone osteocalcin

**DOI:** 10.1002/jcp.28707

**Published:** 2019-04-26

**Authors:** Sophie A. Millar, Susan I. Anderson, Saoirse E. O'sullivan

**Affiliations:** ^1^ Division of Medical Sciences and Graduate Entry Medicine, School of Medicine, Royal Derby Hospital University of Nottingham United Kingdom

**Keywords:** AKT, aortic endothelial cells, ERK, osteocalcin, p70s6k

## Abstract

The purpose of this study was to characterize the direct effects of uncarboxylated osteocalcin (ucOCN) on vascular cell biology in vitro, to assess its potential function in pathophysiological conditions such as atherosclerosis. Human aortic endothelial cells (HAECs) and smooth muscle cells (HASMCs) were treated with ucOCN (0.1–50 ng/ml) and changes in phosphorylation of intracellular signaling proteins, angiogenesis, proliferation, migration, monolayer permeability, and protein secretion were measured. In HAECs, phosphorylated JNK and CREB were decreased with ucOCN (*p* < 0.05). In HASMCs, phosphorylated p70S6K and NF‐ΚB were increased by ucOCN (*p* < 0.05). Cell proliferation increased in both cell types dose dependently which was blocked by AKT and ERK pathway inhibitors. ucOCN did not affect cell permeability, angiogenesis, or migration. The direct activity of ucOCN on vascular cells is recognized, particularly its proliferative effects. However, at least in physiological settings, it does not appear that osteocalcin may directly promote atherogenesis based on the outcomes measured.

**Abbreviations**
OCN: osteocalcin
ucOCN: uncarboxylated osteocalcin
HAEC: human aortic endothelial cell
HASMC: human aortic smooth muscle cell

## 1 INTRODUCTION

In recent years, the skeleton's role as an endocrine organ has been increasingly acknowledged and investigated, in particular its cross‐talk with glucose and lipid metabolism (Ferron & Lacombe, 2014; Lee et al., 2007). These links have been attributed in part to a vitamin K dependent protein called osteocalcin (OCN), also known as bone Gla protein or BGLAP. OCN is the most abundant noncollagenous protein found in the bone matrix, predominantly produced by osteoblasts (Hauschka, Lian, Cole, & Gundberg, 1989). Posttranslational γ‐carboxylation of glutamic acid residues results in a carboxylated form of OCN (cOCN) which induces a high affinity for calcium ions. This promotes binding of cOCN to hydroxyapatite crystal lattices present in the bone extracellular matrix. Uncarboxylated osteocalcin (ucOCN) is released into the circulation, as well as some cOCN, following a number of conditions such as decarboxylation, vitamin K deficiency, or by the acidic environment during bone resorption (Cairns & Price, 1994; Motyl, Mccabe, & Schwartz, 2010; Plantalech, Guillaumont, Vergnaud, Leclercq, & Delmas, 1991).

OCN has an established extra‐skeletal function through involvement with whole body metabolism, fertility, and cognition (Ferron et al., 2010; Hauschka et al., 1989; Lee et al., 2007; Oury et al., 2011, 2013), reviewed elsewhere (Karsenty, 2017). A new line of enquiry into the wide‐ranging function of OCN is its role in the vasculature, and it has been proposed as a biomarker for cardiometabolic risk, and a potential therapeutic target (Tacey et al., 2018). It has been reported that in older men, a lower ratio of ucOCN/total OCN predicted myocardial infarction independently of conventional cardiovascular risk factors (Yeap et al., 2015). However, other studies have found no association between OCN and cardiovascular disease incidence (Holvik et al., 2014; Hwang et al., 2015). Elsewhere, Yeap et al. (2012) found that total osteocalcin predicted all‐cause mortality and cardiovascular disease‐related mortality in community‐dwelling older men, however the relationship was U‐shaped with men at both ends of the distribution at increased risk. Similarly, Kanazawa, Yamaguchi, and Sugimoto (2011) performed a longitudinal study in which a U‐shaped association between OCN and plaque score was determined. It appears OCN may protect vascular function, which is often mediated through an improved metabolic state involving insulin signaling and glucose regulation (Dou et al., 2014; Jung et al., 2013; Tacey et al., 2018). *In vivo* studies in rats and mice found that ucOCN may be protective against atherosclerosis and promotes normal vascular function (Tacey et al., 2018). Direct effects of OCN on vascular cells remains to be confirmed through *in vitro* investigation. Other bone hormones such as FGF‐23, and more recently sclerostin, have already been demonstrated to influence vascular cells directly (Cianciolo et al., 2018; Oranger et al., 2017).

Another particular area of interest is the relationship between OCN and vascular calcification. This is based on the underlying resemblance of vascular calcification to bone mineralization and limited direct investigations with osteocalcin and the vasculature (Evrard, Delanaye, Kamel, Cristol, & Cavalier, 2015; Idelevich, Rais, & Monsonego‐Ornan, 2011; Millar, Patel, Anderson, England, & O'sullivan, 2017). Cross‐sectional epidemiological data surrounding osteocalcin has reported conflicting associations and a meta‐analysis of data in humans correlating OCN and markers of calcification and atherosclerosis was inconclusive (Millar et al., 2017).

*In vitro* examination of OCN in human cells and exploration of its potential role and mechanisms of actions is needed as our knowledge is remarkably limited. To date, it has been reported that ucOCN is the active form of OCN involved in its endocrine functions (Lacombe & Ferron, 2015). Therefore, we undertook comprehensive *in vitro* experiments in human aortic endothelial cells (HAECs) and human aortic smooth muscle cells (HASMCs), assessing their response to ucOCN, with the hypothesis that it can initiate direct intracellular signaling, and promote angiogenesis. We aimed to report ucOCN related intracellular pathways and cellular functions to progress understanding of its importance under normal physiological conditions, and any indications that ucOCN may be a promoter or suppressor of normal vascular function. It is important to explore the effects of ucOCN on both HAECs and HASMCs as each cell type has their distinct characteristics and role in maintaining vascular function and homeostasis, and equally have their own responses and involvement in vascular pathologies.

## 2 MATERIALS AND METHODS

### 2.1 Materials

Human uncarboxylated osteocalcin (ucOCN; amino acids 1–49, [Glu17,21,24]) was purchased from US Biological (O8060‐09C‐USB; Ely, UK) and AnaSpec Inc. (AS‐65307; Ely, UK). The amino acid sequence of purchased osteocalcin was as follows: Tyr‐Leu‐Tyr‐Gln‐Trp‐Leu‐Gly‐Ala‐Pro‐Val‐Pro‐Tyr‐Pro‐Asp‐Pro‐Leu‐Glu‐Pro‐Arg‐Arg‐Glu‐Val‐Cys‐Glu‐Leu‐Asn‐Pro‐Asp‐Cys‐Asp‐Glu‐Leu‐Ala‐Asp‐His‐Ile‐Gly‐Phe‐Gln‐Glu‐Ala‐Tyr‐Arg‐Arg‐Phe‐Tyr‐Gly‐Pro‐Val.

### 2.2 Cell culture

HAECs and HASMCs were purchased from PromoCell (UK) and maintained at 37°C in a humidified incubator supplemented with 5% CO_2_ in commercially available endothelial cell growth media and smooth muscle cell growth media (PromoCell), containing 1% Penicillin–Streptomycin (Sigma‐Aldrich, UK). Cells were used between passages 3 and 5. Human ovarian cancer cell line, SKOV‐3 (American Type Culture Collection [ATCC] HTB‐77) obtained from ATCC were cultured in Roswell Park Memorial Institute‐1640 media (Sigma‐Aldrich) containing 10% fetal bovine serum (FBS; Sigma‐Aldrich) and 1% Penicillin–Streptomycin. SKOV‐3 cells (passage 21) were used as a negative control when identifying the GPRC6A receptor during western blotting. Human osteoblasts (HOBs) were originally isolated from human femoral head trabecular bone and have been characterized previously (Anderson, Downes, Perry, & Caballero, 1998; Henstock, Ruktanonchai, Canham, & Anderson, 2014; Huang, Silvio, Wang, Tanner, & Bonfield, 1997). HOBs were cultured in Dulbecco's Modified Eagle's Medium supplemented with 10% FBS, 1% Penicillin–Streptomycin, 200 nM l‐glutamine, and 15 µg/ml ascorbic acid (all Sigma‐Aldrich) and were used as a positive control for measuring osteocalcin secretion from cells by enzyme‐linked immunosorbent assay (ELISA) and for GPRC6A receptor identification during western blotting. After experimental treatments, cell media was collected and cells were washed with phosphate buffered saline (PBS; pH 7.4, Gibco™, Loughborough, UK). Radioimmunoprecipitation assay buffer (Sigma‐Aldrich) with protease and phosphatase inhibitors (A32959; Thermo Fisher Scientific, Loughborough, UK) was added to lyse the cells which were then collected and centrifuged at 14,000*g* for 5 min at 4°C. Cell supernatants were frozen at − 80°C or analyzed immediately, unless otherwise stated.

### 2.3 Vascular permeability

HAECs were grown until confluent in 12‐well plates on 12 mm diameter, 0.4 µM pore polycarbonate membrane inserts (Corning® Costar®; Sigma‐Aldrich). Transepithelial electrical resistance (TEER) was measured using EVOM™ voltohmmeter (World Precision Instruments, Sarasota, FL) to evaluate paracellular permeability of cells treated with vehicle or ucOCN (10 ng/ml).

### 2.4 Enzyme‐linked immunosorbent assays

Human osteocalcin DuoSet ELISA (R&D Systems; DY1419) was used to measure total secreted osteocalcin in cell culture media. Endothelin Pan Specific, ICAM‐1/CD54, VCAM‐1/CD106, and total MMP‐3 DuoSet ELISAs were performed on cell culture media according to the manufacturer's instructions (Catalog numbers DY1160, DY720, DY809, and DY513; R&D Systems, Abingdon, UK).

### 2.5 Proliferation assay

Cell Titre 96 AQueous One Solution Cell Proliferation Assay (Catalog No. G3581; Promega, Southampton, UK) was performed according to manufacturer's instructions in HAECs and HASMCs. Cells were seeded at approximately 5,000 cells per well in 96‐well plates and left to adhere for at least 4 hr. Cells were then treated with control media, or media supplemented with 0.1 ng/ml, 0.5 ng/ml, 1 ng/ml, 10 ng/ml or 50 ng/ml of ucOCN for 48 or 72 hr. The proliferation assay was repeated with 10 ng/ml of ucOCN on confluent cells to establish if the effects of OCN were due to an increase in cell proliferation as opposed to an increase in cell metabolism.

The proliferation assay was repeated using 10 ng/ml of ucOCN alone or in the presence of 3 µM PI3K inhibitor (LY294002 hydrochloride; Catalog No. 1130; Tocris Bioscience, Bristol, UK) or MAPK (MKK/MEK) inhibitor (PD98059; Catalog No. 1213; Tocris Bioscience).

### 2.6 Scratch wound/migration assay

An *in vitro* scratch assay with HAECs and HASMCs was performed as described previously (Liang, Park, & Guan, 2007). Cells were seeded in six‐well cell culture plates and left to adhere until confluent in usual cell growth media. For the HAEC experiments, cells were then sustained with endothelial cell basal medium (PromoCell) without growth or supplement factors but containing 10% FBS (F9665, Sigma‐Aldrich) to allow cell survival but limiting proliferation. For the HASMC experiments, once confluent, cells were switched to smooth muscle cell basal medium (PromoCell) without growth or supplement factors but supplemented with 0.1% FBS. After 24 hr, a scratch was marked on each well with a P200 pipette tip, washed with PBS, and replaced with media alone, or media containing ucOCN (10 ng/ml). Images of the scratch were captured at baseline and at various time points to monitor cell migration. The area of the scratch wound at baseline and at each time point was measured using the ImageJ software.

### 2.7 Angiogenesis assay

Confluent (80–100%) or sub‐confluent (50–60%) HAECs were treated with ucOCN (10 ng/ml) or with media alone for 72 hr. The MILLIPLEX MAP Human Angiogenesis Assay (Catalog No. HAGP1MAG‐12K; Merck Millipore, Hertfordshire, UK) was performed according to the manufacturers’ instructions to detect changes in angiopoietin‐2 (ANG‐2), vascular endothelial growth factor d (VEGFd), hepatocyte growth factor (HGF), VEGFc, interleukin‐8 (IL‐8) and fibroblast growth factor‐2 (FGF‐2) in cell lysates.

### 2.8 GPRC6A expression

HAECs, HASMCs, SKOV‐3s, and HOBs were grown until confluent and cultured for an additional 24 hr. Cell lysates were collected and protein samples (5 µg/lane) were resolved by electrophoresis on 10% Mini‐protean TGX precast gels (Bio‐Rad Laboratories, Inc., Hertfordshire, UK). The proteins were wet transferred to a nitrocellulose membrane and incubated in blocking buffer (2% bovine serum albumin in tris buffered saline with tween 20 [TBST]) overnight at 4°C. The membrane was then incubated with rabbit anti‐human GPRC6A primary antibody (Abbexa, Cambridge, UK; 1:1,000 in 3% blocking buffer) for 2 hr at room temperature. The membrane was then washed and incubated for 1.5 hr with alkaline phosphatase conjugated antirabbit secondary antibody (Catalog No. A3937, 1:25,000; Sigma‐Aldrich). Immunoreactive bands were visualized by chemiluminescence (Bio‐Rad Immun‐Star™ AP Substrate Pack #1705012). Protein bands were visualized using the ChemiDoc™ MP Imaging system with Image Lab™ software (Bio‐Rad Laboratories, Inc.). The membrane was then stripped for reblotting using ReBlot Plus Mild Antibody Stripping Solution (Sigma‐Aldrich) and then blocked with 5% marvel in TBST for 2 hr at room temperature. The membrane was then incubated with rabbit anti‐human beta‐actin (Abcam, Cambridge, UK; Catalog No. 8227, 1:5,000 in 3% marvel in TBST) overnight at 4°C. Finally, the membrane was washed and incubated with secondary antibody in 3% marvel in TBST and visualized and quantified as above.

### 2.9 Cell signaling assay

HAECs and HASMCs were grown until confluent and treated with ucOCN (10 ng/ml) or with control media for 2, 5, 10, and 30 min. The MILLIPLEX MAP 9‐plex Multi‐Pathway Magnetic Bead Signaling Kit (48‐680MAG; Merck Millipore) was performed according to the manufacturers’ instructions to detect changes in phosphorylated ERK/MAP kinase 1/2 (Thr185/Tyr187), AKT (Ser473), STAT3 (Ser727), JNK (Thr183/Tyr185), p70s6 kinase (Thr412), NF‐kB (Ser536), STAT5A/B (Tyr694/699), CREB (Ser133), and p38 (Thr180/Tyr182) in cell lysates using Luminex® xMAP® technology. Phosphorylated‐ and total‐mTOR (Ser2448) were also measured in the lysate samples using the Luminex system (48‐625MAG, Merck Millipore).

### 2.10 Protein content assay

A bicinchoninic acid protein (BCA) assay was performed to quantify the total protein content in cell lysates (Smith et al., 1985). The BCA working reagent was prepared by mixing BCA solution with copper (II) sulfate pentahydrate 4% solution (Sigma‐Aldrich) at a 50:1 ratio. Protein concentrations of samples were interpolated against a bovine serum albumin standard curve. Secreted protein concentrations, intracellular signaling proteins, and intracellular angiogenesis markers were corrected for total protein content unless otherwise indicated.

### 2.11 Statistical analysis

For cell signaling analyses, two‐way analysis of variances (ANOVAs) were performed using treatment (ucOCN or control) and time point (2, 5, 10, and 30 min) as factors. One‐way ANOVAs were performed to detect differences in protein secretions and angiogenesis markers between treatment groups. A one‐way ANOVA was used to detect differences in cell viability between groups. Results are presented as mean % change to control and standard error of the mean. Multiple comparisons were adjusted for by Dunnett's or Sidak's statistical hypothesis test. All statistical analyses were performed using Prism 7 for Windows (Version 7.01, GraphPad Software Inc.). Adjusted *p* values were considered significant at *p* < 0.05.

## 3 RESULTS

### 3.1 Osteocalcin directly activates intracellular signaling responses in HAECs and HASMCs

It was found that secreted osteocalcin was not detectable in untreated HAECs or HASMCs following 72 hr of confluence (Figure ​​​​​S1A).

**Figure 1:**
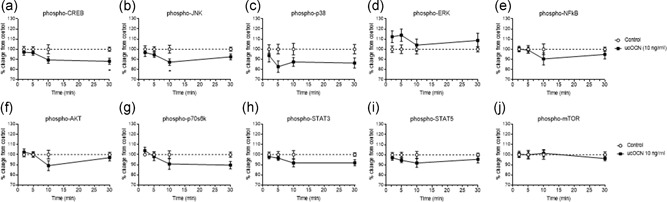
The effects of osteocalcin on human aortic endothelial cell signaling. (a–i) Luminex® xMAP® technology was used to detect changes in phosphorylated CREB (pS133), JNK (pT183/pY185), NFkB (pS536), p38 (pT180/pY182), ERK (pT185/pY187), Akt (pS473), p70 S6K (pT412), STAT3 (pS727), and STAT5A/B (pY694/699) (Milliplex™, 48‐680MAG, Merck Millipore) in cell lysates when treated with ucOCN (10 ng/ml) for 2, 5, 10, and 30 min (*n* = 10 for control and *n* = 20 for ucOCN from three experimental repeats, normalized to total protein). (j) Changes in phosphorylated mTOR in cell lysates were also detected using the Luminex system (*n* = 8 for control and *n* = 16 for ucOCN from three experimental repeats, normalized to total mTOR; Milliplex™, 48‐625MAG, Merck Millipore). Data were analyzed by two‐way ANOVA. Multiple comparisons were adjusted for by Sidak's statistical hypothesis test. Data are given as means with error bars representing SEM. *Denotes a significant difference compared to control (*p* < 0.05). ANOVA: analysis of variance; SEM: standard error of mean; ucOCN: uncarboxylated osteocalcin

In HAECs, treatment with ucOCN reduced phosphorylation levels of CREB at 30 min (*p* < 0.05; Figure 1a). Phosphorylated JNK was decreased at 10 min with ucOCN (*p* < 0.05; Figure 1b). ucOCN appeared to decrease p38 phosphorylation and increase ERK phosphorylation but this did not reach significance (Figure 1c,d). There were no significant differences in phosphorylation of NF‐kB, AKT, p70s6k, STAT3, STAT5, or mTOR with ucOCN treatment compared with control (Figure 1e–j).

**Figure 2:**
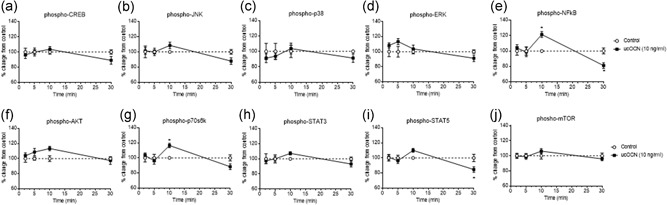
The effects of osteocalcin on human aortic smooth muscle cell signaling. (a–i) Luminex® xMAP® technology was used to detect changes in phosphorylated CREB (pS133), JNK (pT183/pY185), NFkB (pS536), p38 (pT180/pY182), ERK (pT185/pY187), Akt (pS473), p70 S6K (pT412), STAT3 (pS727), and STAT5A/B (pY694/699) (Milliplex™, 48‐680MAG, Merck Millipore) in cell lysates when treated with ucOCN (10 ng/ml) for 2, 5, 10, and 30 min (*n* = 10 for control and *n* = 20 for ucOCN, from three experimental repeats, normalized to total protein). (j) Changes in phosphorylated mTOR in cell lysates were also detected using the Luminex system (*n* = 6 for control and *n* = 12 for ucOCN from three experimental repeats, normalized to total mTOR; Milliplex™, 48‐625MAG, Merck Millipore). Data were analyzed by two‐way ANOVA. Multiple comparisons were adjusted for by Sidak's statistical hypothesis test. Data are given as means with error bars representing SEM. *Denotes a significant difference compared to control (*p* < 0.05). ANOVA: analysis of variance; SEM: standard error of mean; ucOCN: uncarboxylated osteocalcin

In HASMCs, 10‐min treatment with ucOCN increased NF‐kB phosphorylation levels compared to control (*p* < 0.05; Figure 2e). In contrast, after 30 min, ucOCN decreased NF‐kB phosphorylation compared to control (*p* < 0.05; Figure 2e). Phosphorylated p70s6k concentrations were increased at 10 min following ucOCN treatment (*p* < 0.05; Figure 2g). Phosphorylated STAT5 was decreased after 30 min (*p* < 0.05; Figure 2i). Phosphorylated AKT levels appeared increased with ucOCN after 10 min but this did not reach significance (Figure 2f). No significant differences were observed for levels of phosphorylated CREB, JNK, ERK, p38, STAT3, or mTOR with ucOCN treatment compared to control (Figure 2a–d,h,j).

### 3.2 Osteocalcin increases cell proliferation but not migration or angiogenesis markers in HAECs

**Figure 3:**
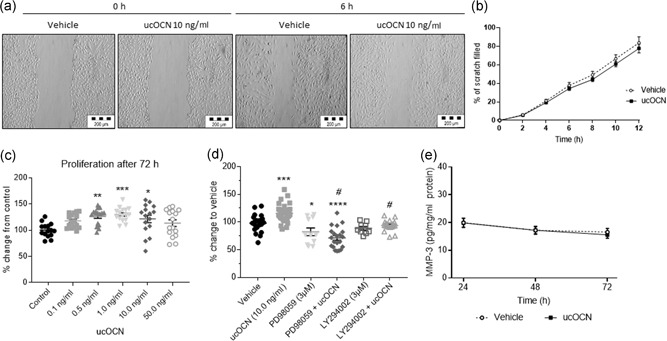
The effects of osteocalcin on human aortic endothelial cell (HAEC) migration and proliferation. (a) Representative images from a scratch wound assay performed in HAECs in which the scratch area was monitored for up to 12 hr. (b) Rate of migration/closure of scratch of HAECs treated with either vehicle or ucOCN (10 ng/ml; *n* = 12 for control and *n* = 24 for ucOCN, from three experimental repeats). (c) ucOCN concentration‐effect (0.1–50.0 ng/ml) on cell proliferation as measured by MTS assay after 72 hr (*n* = 16 from three experimental repeats). Control media and osteocalcin prepared media was replaced after 48 hr. (d) Cell proliferation was further assessed using PI3K inhibitor (LY294002 hydrochloride 3 µM) and MAPK (MKK/MEK) inhibitor (PD98059 3 µM) (three repeats, total at least *n* = 9). (e) MMP‐3 secretion over 24–72 hr by HAECs treated with vehicle or ucOCN (10 ng/ml; *n* = 9 for control and *n* = 18 for ucOCN, from three experimental repeats). Data are given as means with error bars representing SEM. Data were analyzed by one‐way ANOVA and multiple testing corrected for by Dunnet's statistical test. *Denotes a significant difference compared to control (*p < 0.05, **p < 0.01, ***p < 0.001, and ****p < 0.0001). ^#^Denotes significant difference compared to ucOCN. ucOCN, uncarboxylated osteocalcin; MMP‐3, matrix metalloproteinase‐3; MTS: Cell Titre 96 AQueous One Solution Cell Proliferation Assay

**Figure 4:**
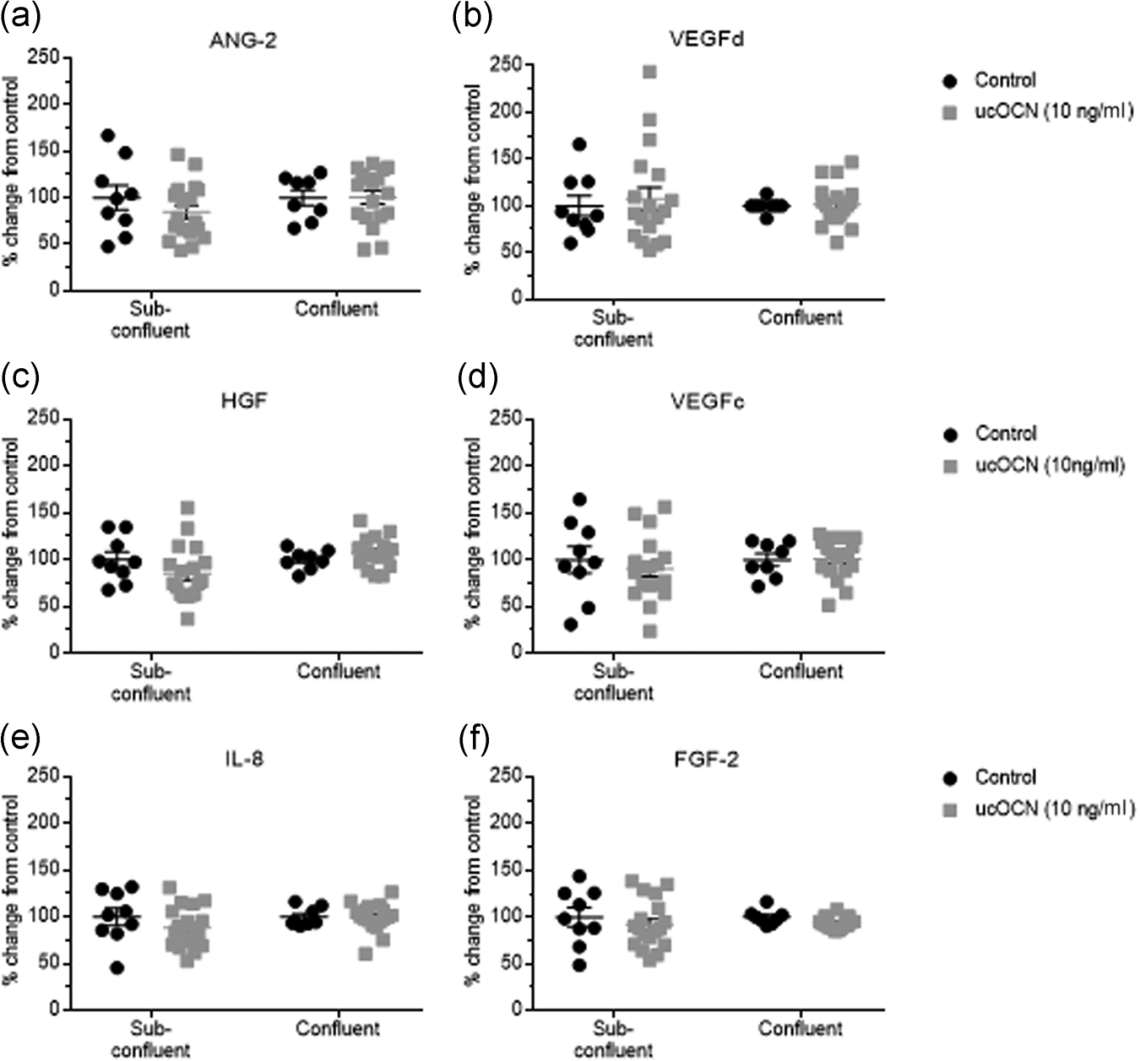
The effects of osteocalcin on markers of angiogenesis in human aortic endothelial cells. (a–f) Luminex® xMAP® technology (Milliplex™, 48‐680MAG, Merck Millipore) was used to detect changes in markers of angiogenesis (angiopoietin‐2, VEGFd, HGF, VEGFc, IL‐8, and FGF‐2) in cell lysates following treatment with ucOCN (10 ng/ml, 72 hr) on subconfluent and confluent cells (*n* = 9 for control and *n* = 18 for ucOCN, from three experimental repeats). In the confluent cells, control media and osteocalcin containing media was replaced every 24 hr. In subconfluent cells, media was not replaced during the 72 hr. ucOCN: uncarboxylated osteocalcin; ANG‐2: angiopoietin‐2; VEGFd: vascular endothelial growth factor d; HGF: hepatocyte growth factor; VEGFc: vascular endothelial growth factor c; IL‐8: interleukin‐8; FGF‐2: fibroblast growth factor‐2

As our results indicated a direct effect of ucOCN in vascular cells, we went on to examine whether OCN influenced stages of angiogenesis of endothelial cells (matrix degradation, proliferation, and migration). There were no significant differences in cell migration/wound closure rates between treatments (control or ucOCN; Figure 3a,b). The potential influence on matrix degradation was assessed by matrix metalloproteinase‐3 (MMP‐3) production after 24–72 hr. MMP‐3 secretion was not affected by ucOCN compared to control (Figure 3e). However, after 72 hr of treatment ucOCN increased proliferation (0.5, 1.0, and 10.0 ng/ml; *p* < 0.05; Figure 3c). The effect of ucOCN on HAEC proliferation was no longer observed when cells were cotreated with AKT or ERK inhibitors (Figure 3d). When a similar experiment was conducted in already confluent cells treated with ucOCN, there were no significant differences between groups, suggesting OCN is not affecting cellular metabolism (Figure S1b,c). In addition, a panel of angiogenesis markers were measured after treatment with ucOCN in confluent and subconfluent cells. After 72 hr of incubation, there were no differences in angiogenic regulators (ANG‐2, HGF, FGF‐2, VEGFc, IL‐8, and VEGFd) between treatment groups in confluent experimental conditions nor in still growing, subconfluence cells (Figure 4a–f).

### 3.3 Osteocalcin increases cell proliferation in HASMCs but not migration

**Figure 5:**
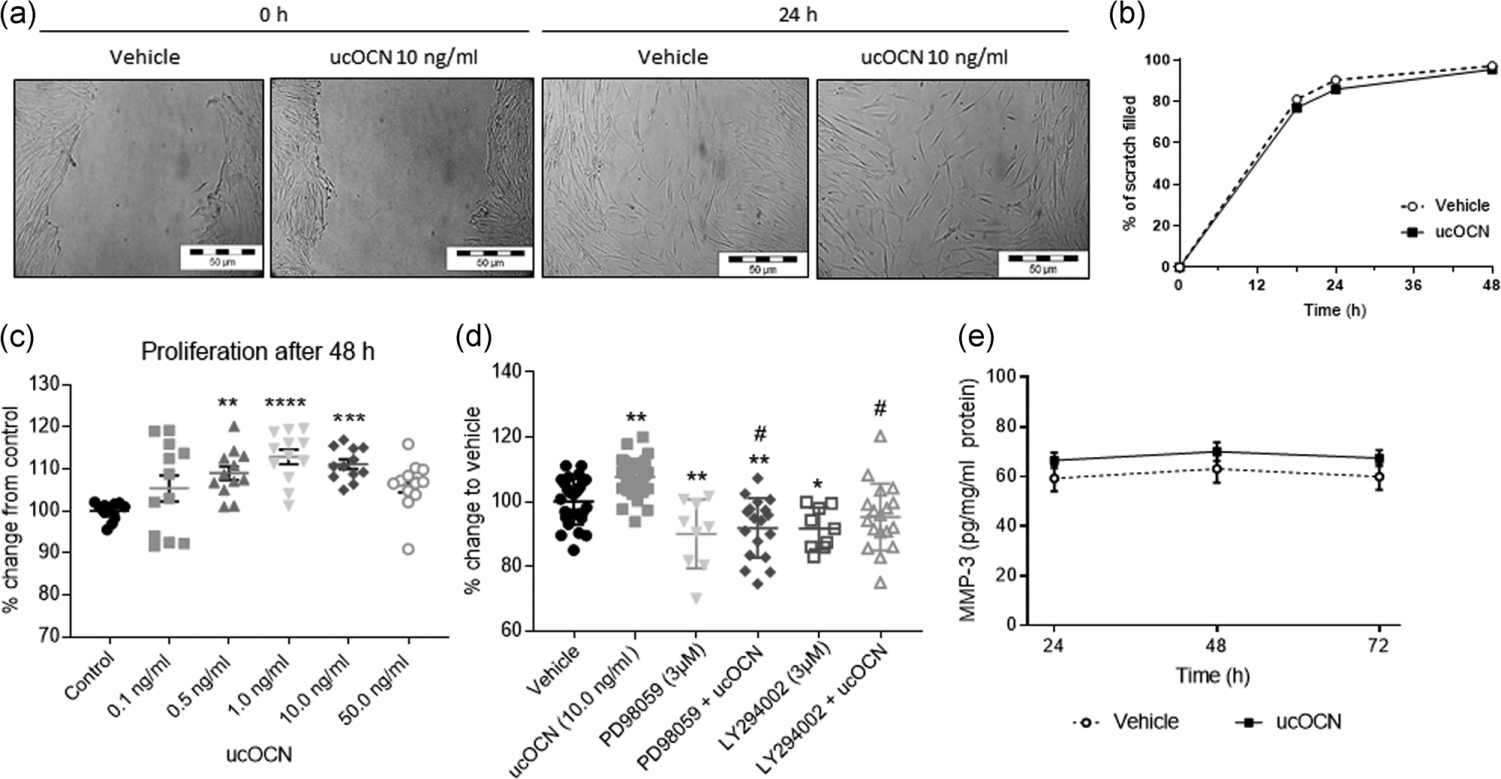
The effects of osteocalcin on human aortic smooth muscle cell (HASMC) migration and proliferation. (a) Representative images from a scratch wound assay performed in HASMCs in which the scratch area was monitored for up to 48 hr. (b) Rate of migration/closure of scratch of HASMCs treated with either vehicle or ucOCN (10 ng/ml; *n* = 23 from four experimental repeats). (c) ucOCN concentration‐dependent effect (0.1–50.0 ng/ml) on cell proliferation as measured by MTS assay after 48 hr (*n* = 12 from three experimental repeats). (d) Cell proliferation was further assessed using PI3K inhibitor (LY294002 hydrochloride) and MAPK (MKK/MEK) inhibitor (PD98059) (three experimental repeats, total at least *n* = 9). (e) MMP‐3 secretion over 24–72 hr by HAECs treated with vehicle or ucOCN (10 ng/ml; *n* = 12 for control and *n* = 24 for ucOCN, from three experimental repeats). One‐way ANOVAs were used to analyze proliferation data and multiple testing corrected for by Dunnett's statistical test. Data are given as means with error bars representing SEM. *Denotes a significant difference compared to control (*p < 0.05, **p < 0.01, ***p < 0.001, and ****p < 0.0001). ^#^Denotes significant difference compared to ucOCN. ANOVA: analysis of variance; SEM: standard error of mean; MMP‐3, matrix metalloproteinase‐3; MTS: Cell Titre 96 AQueous One Solution Cell Proliferation Assay; ucOCN, uncarboxylated osteocalcin

Our results showed that ucOCN did not affect the rate of wound closure (Figure 5a,b). Supporting this, we found ucOCN did not affect production of MMP‐3 after 24–72 hr incubation (Figure 5e). ucOCN (0.5, 1.0, and 10.0 ng/ml) significantly increased cell proliferation after 48 hr compared to control (Figure 5c). This effect was blocked when cells were cotreated with either AKT or ERK pathway inhibitors (Figure 5d).

### 3.4 Osteocalcin does not affect endothelial cell permeability or adhesion makers

**Figure 6:**
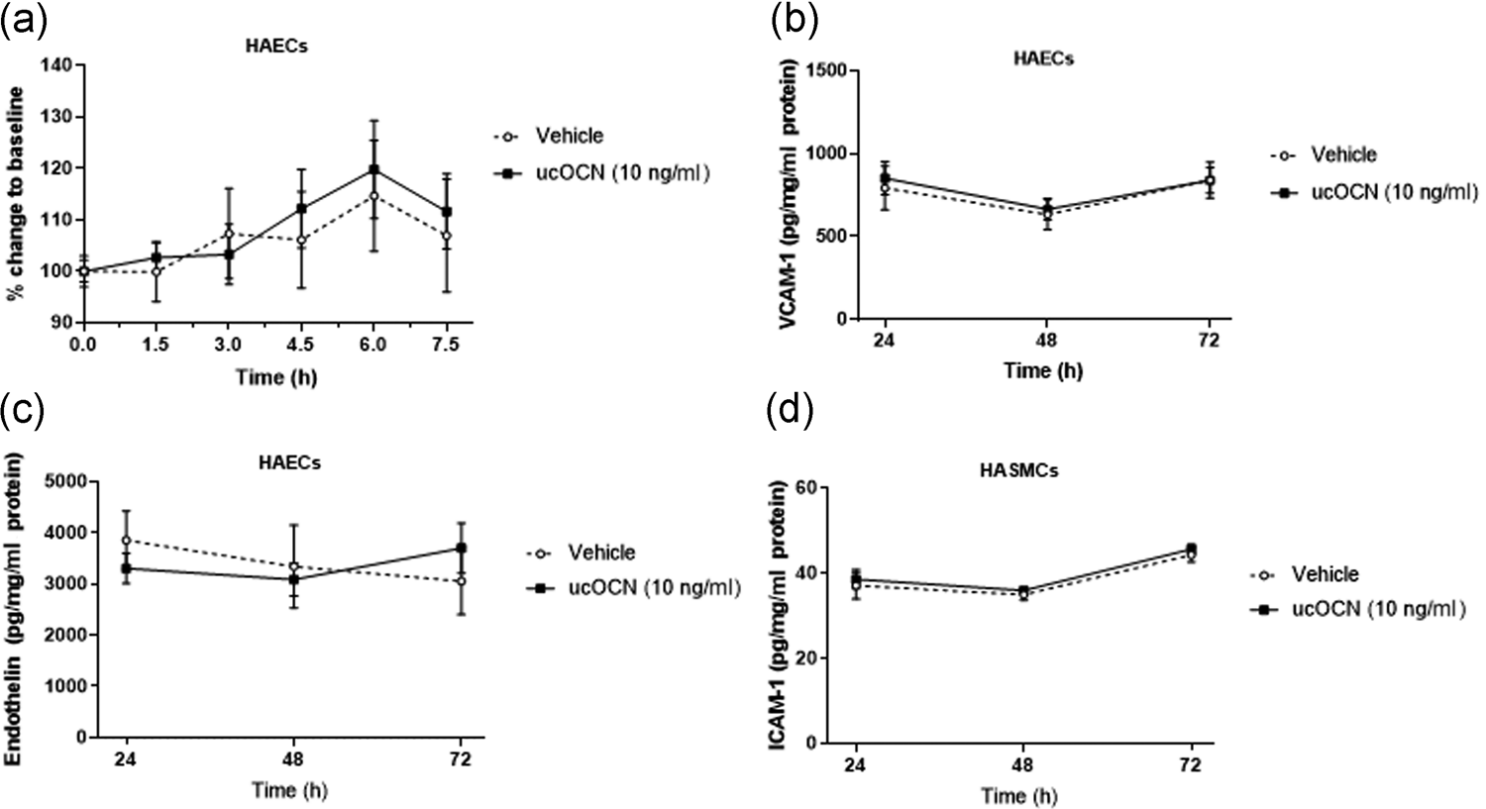
The effects of osteocalcin on vascular cell permeability, endothelin and adhesion markers. (a) Transepithelial electrical resistance measurement was used to evaluate the paracellular permeability of confluent human aortic endothelial cell monolayers treated with or without ucOCN and cOCN (*n* = 10 for control and *n* = 20 for ucOCN, from five experimental repeats). ucOCN (10 ng/ml) did not affect secretion of VCAM‐1 (b) or endothelin‐1 (c) after 24, 48 and 72 hr (*n* = 14 for control and *n* = 28 for ucOCN, from three experimental repeats) from confluent human aortic endothelial cells. (d) Secretion of ICAM‐1 did not differ over 24–72 hr between vehicle or ucOCN (both 10 ng/ml) in confluent human aortic smooth muscle cells (*n* = 12 for control and *n* = 24 for ucOCN, from three experimental repeats). Data are given as means with error bars representing SEM. ICAM‐1: intracellular adhesion molecule‐1; SEM: standard error of mean; ucOCN: uncarboxylated osteocalcin; VCAM‐1: vascular cell adhesion molecule‐2

Acute treatment with ucOCN did not affect endothelial cell permeability, as measured using TEER over 7.5 hr (Figure 6a). Adhesion molecules VCAM‐1 and ICAM‐1 were measured in HAECs and HASMCs respectively. ucOCN did not affect their secretion over time (Figure 6b,d). In addition, ucOCN did not affect production of endothelin, a vasoconstrictor, in HAECs (Figure 6c).

### 3.5 Posited osteocalcin receptor, GPRC6A, is present in HAECs and HASMCs

**Figure 7:**
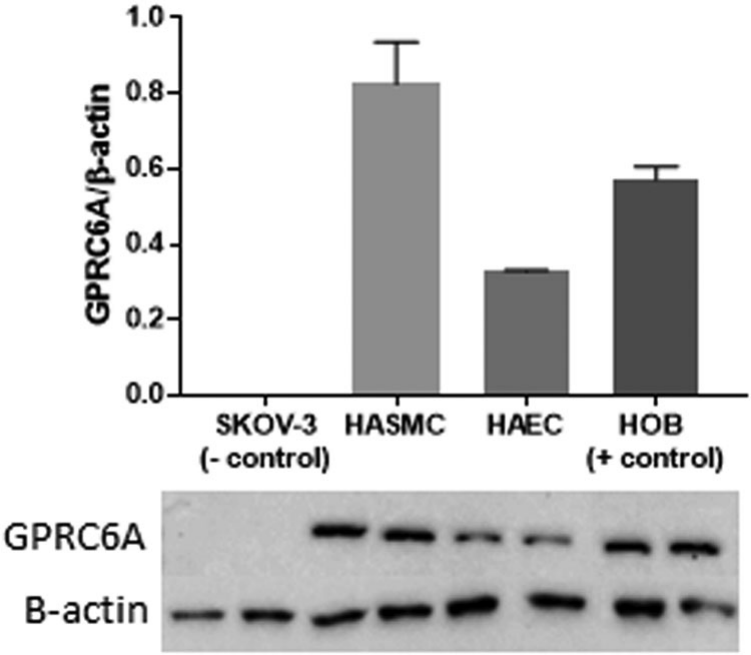
Posited osteocalcin receptor, GPRC6A, is present in human aortic endothelial (HAEC) and smooth muscle cells (HASMC). Western blot data showing lack of presence of GPRC6A in ovarian carcinoma cells (SKOV‐3; negative control), and presence in HASMCs, HAECs, and human osteoblasts (HOBS; positive control). Data are given as means with error bars representing SEM (*n* = 2). SEM: standard error of mean

The proposed osteocalcin receptor, GPRC6A, was detected in HAECs, HASMCs, and HOBs (positive control), and not in SKOVS (negative control) via western blotting (Figure 7).

## 4 DISCUSSION

The aim of this study was to examine the direct vascular effects of physiologically relevant concentrations of osteocalcin (Binkley, Krueger, Engelke, Foley, & Suttie, 2000; Hannemann et al., 2013; Luukinen et al., 2000). ucOCN activated acute cell signaling cascades in HAECs and HASMCs, suggesting there is a molecular target for OCN on these cells. In both cell types, ucOCN increased proliferation through ERK and AKT signaling pathways. OCN does not appear to play a role in migration, endothelial cell angiogenesis or permeability.

In humans, the BGLAP (bone gamma‐carboxyglutamate protein) gene for encoding OCN is ubiquitously expressed in brain, colon, appendix, and many other tissues (NCBI, 2019). However, data on protein expression is less widely available. OCN has been implicated in vascular calcification plaques (Bini, Mann, Kudryk, & Schoen, 1999; Dweck et al., 2014; Foresta et al., 2013; O'Neill & Adams, 2014; Rajamannan et al., 2005), and differentiated osteoblast‐like HASMCs are known to produce OCN during vascular calcification and mineralization (Dhore et al., 2001). Our results found that OCN expression was not detectable in HASMC or HAEC supernatants or intracellularly in normal physiological conditions, suggesting that OCN production from HASMCs is restricted to a pathophysiological setting exclusive to transdifferentiated HASMCs.

ucOCN is found within the circulation although there are no standardized reference values. Total OCN concentrations have been reported by Hannemann et al. (2013) by age and gender. However, reported serum ucOCN concentrations largely varied between <1 and >30 ng/ml (Binkley et al., 2000; Iki et al., 2012; Plantalech et al., 1991; Sowers et al., 1999). We therefore used 10 ng/ml which has also been used in previous *in vitro* studies (Dou et al., 2014; Jung et al., 2013).

We aimed to examine whether exogenously added fully uncarboxylated OCN can directly affect intracellular signaling in HAECs and HASMCs. We found that ucOCN significantly altered phosphorylation levels of numerous signaling proteins including CREB and JNK in HAECs, and NF‐kB and p70s6K in HASMCs. Jung et al. (2013) has previously reported an increase in AKT phosphorylation and eNOS and nitric oxide (NO) production in a PI3‐kinase dependent manner in HAECs when treated with ucOCN for 1 hr (0.3–30 ng/ml; Jung et al., 2013). Overall, these results evidence that there is a direct and rapid intracellular response to osteocalcin in HAECs and HASMCs, likely to be receptor‐mediated.

The G protein‐coupled receptor, class C, group 6, subtype A (GPRC6A) is a recently “de‐orphaned” receptor co‐activated by cations, for example, calcium, and basic L‐α‐amino acids (Clemmensen, Smajilovic, Wellendorph, & Brauner‐Osborne, 2014; Wellendorph & Brauner‐Osborne, 2004). Osteocalcin and testosterone have been proposed to also be agonists to this receptor, which is present in rat arteries (Harno et al., 2008; Oury et al., 2011; Pi, Parrill, & Quarles, 2010; Pi, Wu, & Quarles, 2011). However, others have failed to reproduce and confirm these propositions with osteocalcin (Jacobsen et al., 2013; Rueda et al., 2016). In this paper, we present evidence for the existence of the GPRC6A receptor in both HAECs and HASMCs. Delineation of the specificity and functional response of GPRC6A to OCN in the human vasculature remains to be shown, however there is a lack of a selective receptor antagonist. Attempts at siRNA knock‐down of the receptor may prove fruitful but we did not achieve successful knockdown in our attempts (data not shown). Another receptor, GPR158 has also been recently shown to mediate OCN's effect of cognitive function in mice (Khrimian et al., 2017) which also merits investigation in vascular cells.

Furthermore, we examined whether ucOCN affected stages of angiogenesis such as matrix degradation, migration, and proliferation. ucOCN did not affect MMP‐3 production or wound healing *in vitro*. However, ucOCN was shown to increase proliferation in both HAECs and HASMCs, in a dose‐dependent, bell‐shaped manner, which adds to previously reported antiapoptotic effects of osteocalcin in HAECs (Jung et al., 2013). ucOCN has been previously reported as protective against free fatty acid induced apoptosis and low concentrations of ucOCN (0.3 ng/ml) has been shown to increase markers of pancreatic β‐cell proliferation (Ferron, Hinoi, Karsenty, & Ducy, 2008). We revealed that osteocalcin is working through both AKT and ERK converging pathways to translate the increase in proliferation (Mendoza, Er, & Blenis, 2011). We chose these pathways to examine as it has been previously demonstrated by our group (unpublished data) that OCN increases AKT and ERK phosphorylation at longer time points (e.g. 24 hr) in HASMCs, and another study has also demonstrated increases in AKT phosphorylation in HAECs with OCN (Jung et al., 2013).

It has been previously shown in an *in vivo* chick embryo angiogenesis model that osteocalcin promotes angiogenesis (Cantatore, Crivellato, Nico, & Ribatti, 2005). Interestingly, ucOCN did not affect any of the measured angiogenesis regulators. The discrepancy may be due to the use of human cells in our study, within which the effects of OCN may be less pronounced or nonexistent. Our results may indicate that osteocalcin is working to promote proliferation, at least in HAECs, via mechanisms independent of key angiogenic markers. ucOCN also does not appear to acutely increase mTOR phosphorylation, which is a central regulator of cell growth required for angiogenesis and proliferation. These novel findings suggest that OCN may affect vascular cells in a limited range of function, pertaining to proliferation in normal physiological conditions.

OCN did not affect cell permeability. This is a pivotal finding as cell layer integrity is essential to vascular function and degradation is a key, early event in atherogenesis, plaque formation and cell barrier infiltration. We also demonstrated that ucOCN does not influence two key endothelial cell proteins; endothelin, a potent vasoconstrictor, and VCAM‐1. VCAM‐1 expression is increased during inflammation and by cytokines, and as such we have found no evidence to suggest that osteocalcin is involved in the adhesion of monocytes and other cells to the endothelium, nor acts as a promotor of vasoconstriction. In the aortic smooth muscle cells, ICAM‐1, which functions as a leukocyte recruiter and may be thought of as pro‐inflammatory, was not affected by osteocalcin.

There are several circulating forms of OCN that can be found in the circulation including carboxylated OCN to varying degrees (0–2 carboxylated glutamic acid residues), and N‐terminal fragments (Garnero, Grimaux, Seguin, & Delmas, 1994). It is unknown the extent to which structural differences may play in their biological functions (Li, Zhang, Yang, Li, & Dai, 2016). It is a limitation of the current work that not all present circulating forms could be examined. We recommend *ex vivo* and *in vivo* studies be performed to fully understand the effects of OCN on the vasculature.

The *in vitro* investigations conducted here lay a foundation for further research into the vascular effects of osteocalcin. ucOCN initiates direct responses in human aortic endothelial and smooth muscle cells and is proproliferative. However, the lack of effect of OCN on a range of endpoints such as VCAM‐1, MMP‐3, endothelin‐1, and cell permeability, do not suggest a direct involvement of OCN in atherogenesis or blood vessel disease at least in physiological settings. Further investigations within pathological settings and examination of the biological activity of other various fragments of osteocalcin should be undertaken.

## CONFLICT OF INTERESTS

The authors declare that there are no concflict of interersts.

## AUTHOR CONTRIBUTIONS

S. O. S and S. M. designed the experiments which were carried out by S. M. S. M. wrote the manuscript with input from S. O. S and S. A. S. A. and S. O. S supervised the project.

## DATA AVAILABILITY

The data that support the findings of this study are available from the corresponding author upon reasonable request.

## Supporting information

Supporting information

Supporting information
