# Supplementary figures and images for: Human vascular cell responses to the circulating bone hormone osteocalcin
Source: J Cell Physiol. 2019 Apr 26;234(11):21039–48. doi: 10.1002/jcp.28707 (PMC6767466; doi:10.1002/jcp.28707)

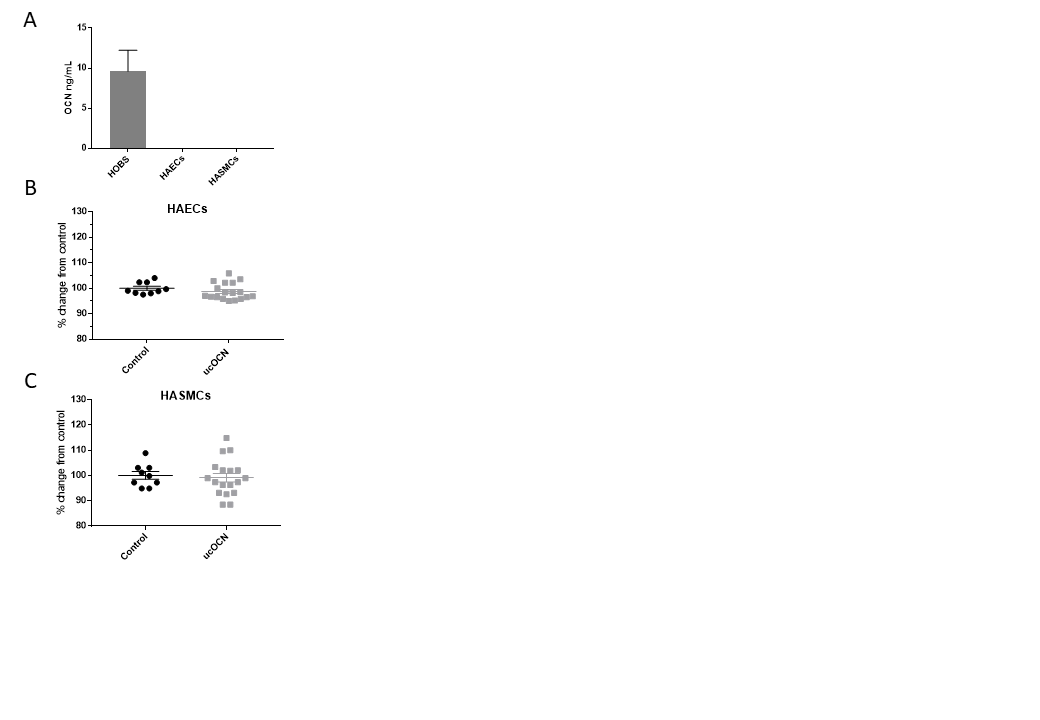

Supplement: Supplementary file 1 — Supporting information [file JCP-234-21039-s001.TIF]
