# Supplementary material for: Human vascular cell responses to the circulating bone hormone osteocalcin
Source: J Cell Physiol. 2019 Apr 26;234(11):21039–48. doi: 10.1002/jcp.28707 (PMC6767466; doi:10.1002/jcp.28707)
Supplement: Supplementary file 2 — Supporting information [file JCP-234-21039-s002.docx]

**Supplementary Figure 1.** Osteocalcin (OCN) secretion detected in human osteoblast cells (HOBS) but not human aortic endothelial cells (HAECs) or human aortic smooth muscle cells (HASMCs) **(A)**. Cell proliferation/MTS assay in confluent HAECs **(B)** and HASMCs **(C)** after treatment with ucOCN (10 ng/mL).
